# Ubiquitin-Associated (UBA) Domain in Human Fas Associated Factor 1 Inhibits Tumor Formation by Promoting Hsp70 Degradation

**DOI:** 10.1371/journal.pone.0040361

**Published:** 2012-08-02

**Authors:** Jae-Jin Lee, Young Mee Kim, Jaeho Jeong, Duk Soo Bae, Kong-Joo Lee

**Affiliations:** 1 Division of Life and Pharmaceutical Sciences, Department of Bioinspired Science, Drug Discovery Research, College of Pharmacy, Center for Cell Signaling, Ewha Womans University, Seoul, Korea; 2 Department of Obstetrics and Gynecology, Samsung Medical Center, SungKyunKwan University, Seoul, Korea; IISER-TVM, India

## Abstract

Human Fas associated factor 1 (hFAF1) is a pro-apoptotic scaffolding protein containing ubiquitin-associating (UBA), ubiquitin like 1 and 2 (UBL1, UBL2), and ubiquitin regulatory X (UBX) domains. hFAF1 interacts with polyubiquitinated proteins via its N-terminal UBA domain and with valosin containing protein (VCP) via its C-terminal UBX domain. Overexpression of hFAF1 or its N-terminal UBA domain significantly increases cell death by increasing the degradation of polyubiquitinated proteins. In this study, we investigated whether hFAF1, whose expression level is reduced in cervical cancer, plays a role in tumor formation. We found that HeLa cells overexpressing full-length hFAF1 or the hFAF1 UBA domain alone, significantly suppressed the anchorage independent tumor growth in soft agar colony formation, increased cell death, and activated JNK and caspase 3. Employing UBA-specific tandem immunoprecipitation, we identified moieties specifically interacting with UBA domain of hFAF1, and found that polyubiquitinated Hsp70s are recruited to UBA domain. We also demonstrated that hFAF1 overexpression promotes Hsp70 degradation via the proteasome. We further found that mutating the UBA domain (I41N), as well as knocking down hFAF1 with specific RNAi, abolishs its ability to increase the proteasomal degradation of Hsp70. These findings suggest that hFAF1 inhibits tumor formation by increasing the degradation of Hsp70 mediated via its UBA domain.

## Introduction

Apoptosis plays a critical role, maintaining homeostasis between cell death and proliferation and is thus a fundamental component in the pathogenesis of cancer. Human Fas-associated factor 1, hFAF1, is a member of the apoptosis signaling complex [1], [2]. hFAF1 enhances Fas-induced apoptosis in murine L-cells, and also initiates apoptosis by itself in BOSC23 cells [2], [3]. hFAF1 also inhibits NFκB activation by binding to p65 subunit and IκB kinase β (IKKβ) [4], [5]. hFAF1 is down regulated in several types of cancer including uterine cervix carcinoma [6] and human gastric carcinomas [7] suggesting that hFAF1 is likely involved in cancer progression. The underlying mechanism, however, is not clear. We previously identified hFAF1 as an ubiquitin receptor, comprising several ubiquitin related domains, UBA, UBL1, UBL2, and UBX [9]. UBA domains recruit polyubiquitinated proteins. UBL1 domain interacts with heat shock protein 70 (Hsp70) [8], and UBX domains bind to valosin-containing protein (VCP), a chaperone of AAA (ATPase associated with different cellular activities) family [9]. Human FAF1 plays key roles in apoptosis through its N-terminal UBA domain by inhibiting protein degradation and causing the accumulation of polyubiquitiated proteins. Expression of hFAF1 is discernibly reduced in cervical cancer tissues, suggesting that it may play an important role in human cancer. In this study, we investigated the molecular mechanism underlying the role of hFAF1 in human cancer, focusing on its function as an ubiquitin receptor.

Ubiquitin-mediated protein degradation is one of the major mechanisms in controlled proteolysis. An enzyme cascade known as activating enzyme (E1)-conjugating enzyme (E2)-ligase (E3) causes the activation and transfer of ubiquitin onto the target protein in a linkage specific manner. Polyubiquitin chains covalently attached to proteins through K48 linkages, are recognized and degraded by the 26S proteasome [10]. The ubiquitin proteasome degradation pathway regulates many cellular activities such as cell cycle regulation, signal transduction, and DNA repair [11], but the mechanism targeting ubiquitinated substrates to the proteasome is not well understood. In recent studies, a wide variety of proteins containing ubiquitin-interacting domains have been identified and their characteristics and roles in various biological processes examined. These studies suggest that each protein containing an ubiquitin-interacting domain serves as an ‘ubiquitin receptor’, which interacts with different ubiquitin chains and substrates, and controls the fates of ubiquitinated substrates, largely depending on the specificity and function of the proteins [12]. The specific substrate requirements for the ubiquitin receptor, which remain to be elucidated, may hold the clues for understanding the cargo systems causing proteasomal degradation [13]. The UBA domain, first identified from bioinformatic analysis, and found in many proteins of the ubiquitin proteasome system (UPS), interacts with various mono- or poly-ubiquitin chains and controls cell cycle control, activates DNA repair and promotes proteasomal degradation [14], [15], [16].

Employing NMR spectroscopy, we recently showed that hFAF1 N-terminal UBA domain binds polyubiquin chains, but not monoubiquitin chains. We also demonstrated by peptide sequencing with tandem mass spectrometry, that hFAF1 mainly interacts *in vivo* with K48 linked polyubiquitin chains [17]. In this study, we employed tandem immunoprecipitation to identify the polyubiquitinated proteins specifically bound to the hFAF1 UBA domain.

The heat shock protein 70 (Hsp70) family plays key roles as molecular chaperones in protein folding, transport and degradation. Members of this family, including Hsp72, are transiently induced in response to various environmental stresses and serve to protect cells against heat shock and other conditions which cause massive damage and protein denaturation [18]. Endogenous expression of Hsp70 which is low in normal conditions, increases in stressed conditions and decreases to basal level within 24 h. How the levels of the newly synthesized Hsp70 decrease to basal level is not clear. We previously showed that hFAF1 interacts with Hsp70 and decreases its chaperone activity and that overexpression of hFAF1 induces pro-apoptotic property [8]. Because of its multiple ubiquitin-related domains and interactions with various proteins including polyubiquitinated proteins such as Hsp70 and VCP, what role hFAF1 plays in tumor formation and how it regulates polyubiquitinated proteins become important questions. We addressed these questions, employing tandem immunoprecipitation techniques combined with mass spectrometric analysis. We found that hFAF1 recruits polyubiquitinated Hsp70 through its N-terminal UBA domain, and that transient overexpression of hFAF1 results in the degradation of Hsp70 through the UBA domain. We therefore hypothesize that hFAF1 regulates cell death and tumor formation by regulating the turnover of Hsp70, one of specific polyubiquitinated substrate proteins.

## Results

**Figure 1:**
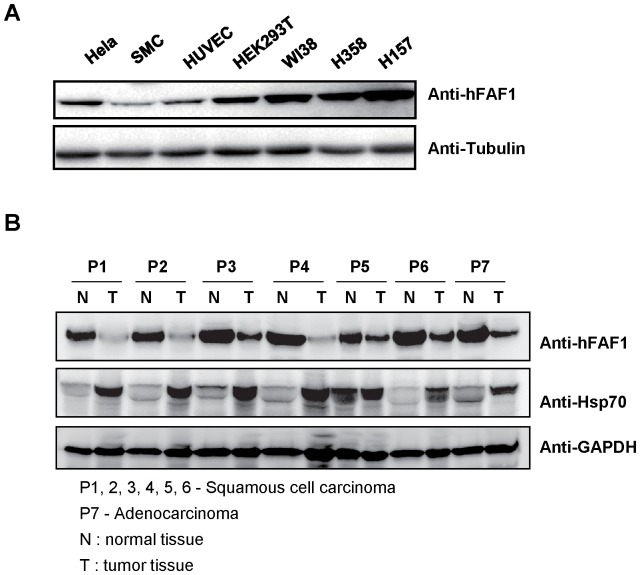
Expression levels of hFAF1 and Hsp70 in cervical cancer tissues. (A) Expression levels of hFAF1 in various cell lines were examined by Western analysis using anti-FAF1 antibody. Tubulin was used as a loading control. (B) hFAF1 levels were specifically reduced in human cervical cancers. Human cervical cancer tissues and corresponding normal tissues were homogenized in a lysis buffer. Homogenized tissues were centrifuged and equal amounts of proteins from the supernatants were loaded in SDS-PAGE. hFAF1 and Hsp70 were detected with specific antibodies followed by HRP-conjugated secondary antibody. GAPDH was used as loading control among the samples.

To characterize the function of hFAF1 in human cancers, we first examined its expression in human cervical cancer tissues. Cervical cancer tissues from seven patients were collected, washed with red blood cell (RBC) lysis buffer, homogenized, and equal amount of proteins were loaded on SDS-PAGE and expression levels of hFAF1 were determined by western analysis. Fig. 1A shows the ubiquitous expression of hFAF1 is in various cell lines. Fig. 1B shows that the expression of hFAF1 is significantly reduced in six squamous cell carcinomas and one adenocarcinoma compared to normal cervical tissues. Because it has been reported that Hsp70s, chaperones that refold denatured proteins, significantly increase in tumor tissues [18], we investigated the effect of hFAF1 expression on tumor formation.

### Overexpression of hFAF1 suppresses colony formation via its UBA domain

**Figure 2:**
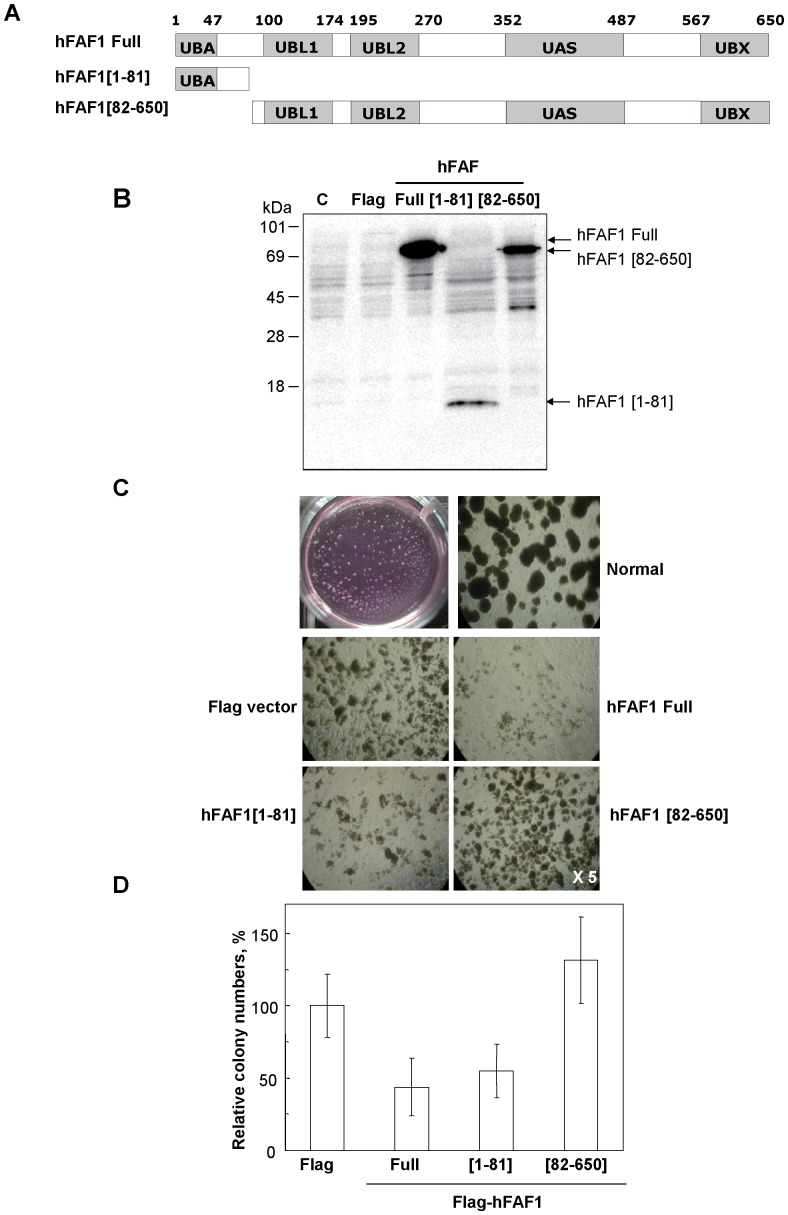
Overexpression of hFAF1 suppresses colony formation through its UBA domain. HeLa cells were transfected with various expression plasmids (A) including pFlag-CMV-2-hFAF1 full, pFlag-CMV-2-hFAF1[1–81], and pFlag-CMV-2-hFAF1[82–650], recovered for 24 h in MEM without antibiotics, and then harvested or seeded in soft agarose gel. (**B**) Protein expression levels were determined on 15% SDS-PAGE. Overexpressed hFAF1 full and truncated forms ([1–81], [82–650]) were detected with hFAF1 specific antibody. (**C**) 10^6^ cells/mL were seeded in soft agarose gel, and then incubated for about 2∼3 weeks for colony formation at 37°C as described in Materials and Method. The images were acquired using light microscopy fitted with digital camera. (**D**) The colonies were counted and data was presented as ± SD. All measurements were done at least in triplicate.

We previously showed that overexpression of hFAF1 causes the accumulation of polyubiquitinated proteins similar to proteasome inhibitors [9]. To determine the relationship between hFAF1 and cancer, we examined colony formation in soft agar, of cells overexpressing various mutants of hFAF1 and whether polyubiquitinated protein recruitment by hFAF1 through its UBA domain affects cancer formation. HeLa cells were transiently transfected respectively with Flag vector control, Flag-hFAF1, Flag-hFAF1[1–81] which contains only the UBA domain, and Flag-hFAF1[82–650] which has no UBA domain, as listed in Fig. 2A. Protein expression levels were determined on 15% SDS-PAGE (Fig. 2B), and soft agar colony formations of these cells were monitored (Fig. 2C) and the results quantified (Fig. 2D). Colony formation was dramatically reduced in cells overexpressing full-length hFAF1 as well as hFAF1[1–81] which contains only the UBA domain. On the other hand, colony numbers and sizes increased in cells transfected with hFAF1[82–650] which has no UBA domain, compared to control vector. These results suggest hFAF1 suppresses colony formation, and that the UBA domain is the moiety responsible for suppressing colony formation.

### Overexpression of hFAF1 induces cell death through its UBA domain

**Figure 3:**
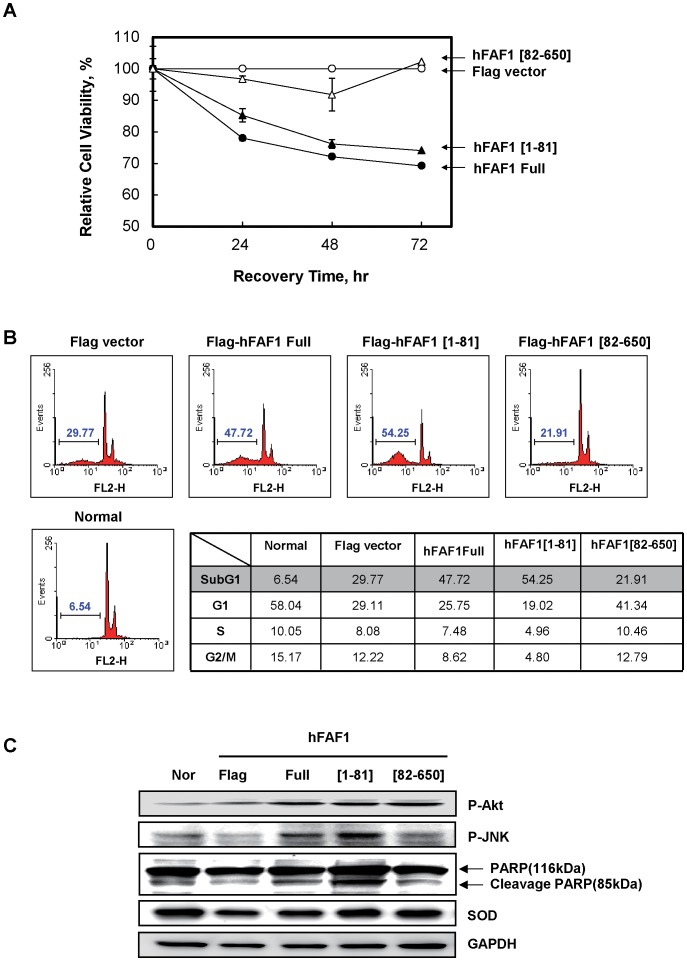
Overexpression of hFAF1 induces cell death through its UBA domain. (**A**) HeLa Cells were transfected with hFAF1 clones for indicated times (24, 48 and 72 h) and cell viability analyzed by MTT assay. (**B**) The transfected cells were stained with PI and cell population at each cell cycle stage was detected by FACS, and data analyzed using Mod-Fit program. (**C**) hFAF1 affected-signaling molecules (phospho-Akt, phospho-JNK, PARP, SOD) were detected by western blot analysis in cells 72 h after transfection. All measurements were done at least in triplicate.

To determine whether reduction of colony formation resulted from cell death or cell growth arrest, we examined apoptosis of HeLa cells expressing various mutants of hFAF1. HeLa cells were transiently transfected with full-length hFAF1 and various truncated Flag-hFAF1s: Flag-hFAF1, Flag-hFAF1[1–81], and Flag-hFAF1[82–650]. Cell viabilities were determined using the MTT assay at 24, 48 and 72 h after transfection. Overexpression of full-length hFAF1 and hFAF1[1–81] decreased cell viability compared to control vector, while overexpression of hFAF [82–650] did not affect cell viability (Fig. 3A). These cell viability changes were confirmed by FACS analysis. Apoptosis was assessed by measuring propidium iodide stained apoptotic body by FACS analysis. Sub-G1 fractions (representing apoptotic cells) significantly increased in cells overexpressing both full-length hFAF1 and truncated hFAF1[1–81], containing the UBA domain, while no discernible changes were observed in cells overexpressing hFAF1[82–650], the UBA domain deleted construct, compared to control (Fig. 3B). The results suggest that both apoptosis and cell death induced by hFAF1 are related to recruitment of polyubiquitinated proteins through UBA domain.

**Figure 4:**
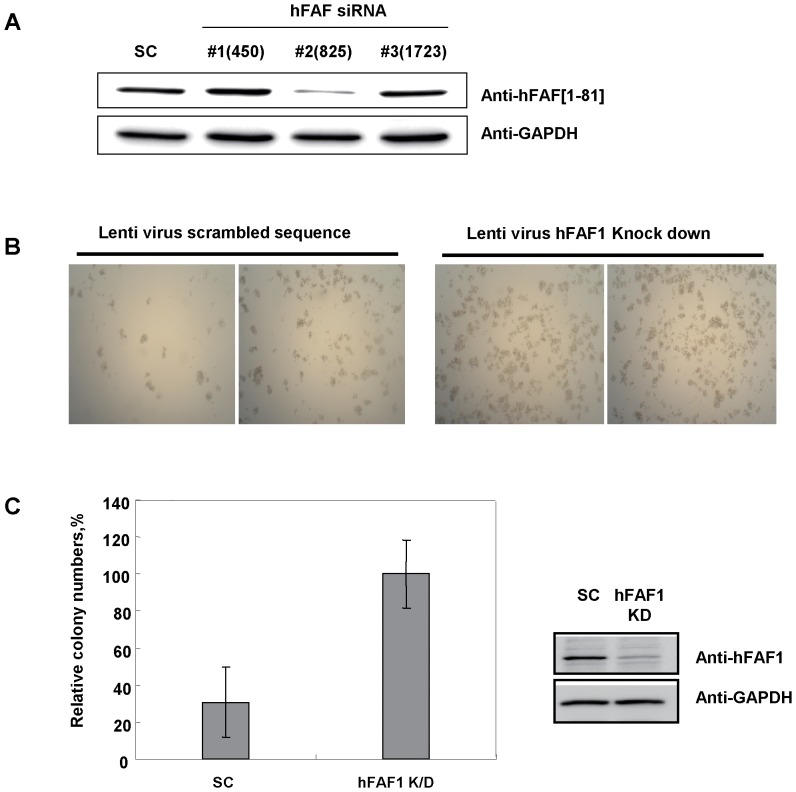
Cells in which hFAF1 was knocked-down are proliferative compared to control cells. (**A**) hFAF1 knock down in cells was performed with Lenti-virus hFAF1 siRNA. hFAF1 siRNA expressing cells were detected by X-gal staining, and the knock-down of hFAF1 was confirmed by western blot analysis using hFAF1 [1–81] truncated protein specific antibody. (**B**) Colony formation significantly increased in cells in which hFAF1 was knocked-down. HeLa cells containing scramble sequences, hFAF1 siRNA #2: HeLa containing hFAF1 specific siRNA #2 sequences were used for colony formation assay. (**C**) Quantitative analysis of colony formation (4B right panel); and the degree of knock down in hFAF1 in stable cells (4B left panel).

To understand how full-length hFAF1 and its [1–81] truncated mutant regulate cell death, we examined the activation of signaling molecules involved in cell proliferation or death, including Akt, JNK, caspase3 substrate PARP cleavage, and ROS related SOD. Activation of Akt, JNK, caspase3 and SOD in cells overexpressing full-length hFAF1, hFAF1[1–81] and hFAF1[82–650] are shown in Fig. 3C. Akt, a molecule downstream of PI3K, plays roles in cell proliferation, cell morphology and cell migration. No discernible changes were observed in Akt activations (phospho-Akt at Ser473) in the cells expressing various hFAF1 mutants. Activation of JNK (phospho-JNK at T183/T185) is required for stress-induced apoptosis. Cells overexpressing full-length hFAF1 and hFAF1[1–81] significantly activated JNK, but no changes were seen in cells expressing hFAF1[82–650]. It is known that caspase 3, activated in response to cell death, cleaves its substrate, PARP. Cells expressing full-length hFAF1 and hFAF1[1–81] induced the cleavage of PARP. These results suggest that full-length hFAF1 and [1–81] truncated hFAF1 induce cell death by activating JNK and caspase 3 through the UBA domain. Since it has been reported that up-regulation of SOD induces the accumulation of intracellular superoxide anion which is very reactive and may cause cellular toxicity, we investigated whether full-length hFAF1 and its truncated mutants induce up-regulation of SOD. We did not detect any accumulation of SOD in hFAF1 transfected cells (Fig. 3C). To investigate whether hFAF1 specifically induces cell death, we developed a stable cell line in which hFAF1 was knocked down with Lenti-virus hFAF1 siRNA. hFAF1 siRNA expression in the cells was detected by X-gal staining, and the knock-down of hFAF1 was confirmed by western blot analysis using antibody specific to truncated hFAF1 [1–81] (Fig. 4A). To confirm that hFAF1 plays a role in tumor suppression, we examined the effect of knocking-down hFAF1 on colony formation in HeLa cells. We found that reduced expression of hFAF1 by Lenti virus significantly increased the colony numbers as shown in Fig. 4B and 4C. These results indicate that hFAF1 plays a role in suppression of tumor formation.

### Identification of polyubiquitinated proteins interacting with UBA domain in hFAF1

**Figure 5:**
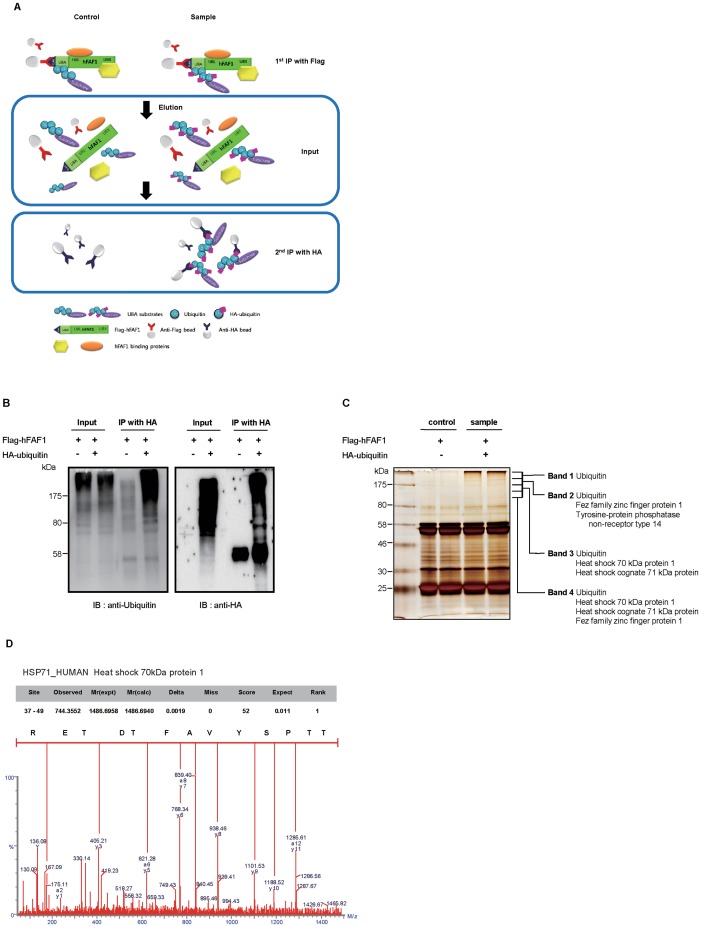
Identification of polyubiquitinated proteins binding to UBA domain of hFAF1. (A) Schematic diagram of UBA domain specific tandem immunoprecipitation of hFAF1. HEK293T cells were co-transfected with Flag-hFAF1 and HA-ubiquitin (or HA-vector). Control cells were co-transfected with Flag-hFAF1 and HA vector, and examined cells were co-transfected with Flag-hFAF1 and HA-ubiquitin. In the first immunoprecipitation, cell lystes were immunoprecipitated with anti-Flag M2 agarose-linked affinity beads. In these precipitates, hFAF1 interactomes via various domains (UBL1 and UBX) were appeared as well as polyubiquitinated proteins bound to UBA domain. Precipitates were eluted by 1% SDS elution buffer and 10x diluted. Second immunoprecipitation with HA affinity beads was performed at room temperature with diluted supernatant. In this IP, only ubiquitinated proteins bound to hFAF1 were precipitated. (B) Second immunoprecipitation with anti-HA antibody was confirmed by Western analysis using anti-ubiquitin antibody. (C) Immunoprecipitated samples were separated in 10% SDS PAGE, and gel silver stained. High molecular weight ubiquitinated proteins binding to hFAF1 were analyzed using UPLC-ESI-q-TOF tandem MS. (D) Hsp70 detected in high molecular weight bands (2–4) were identified by MS/MS as shown by the spectra with K-48 linked ubiquitin chain.

To determine how the UBA domain in hFAF1 mediates apoptosis and suppresses colony formation, we focused on the polyubiquitinated proteins interacting with the UBA domain. An one-step immunoprecipitation procedure did not allow such identification because of the fact that hFAF1 contains multiple domains interacting with various proteins including Hsp70, VCP and other polyubiquitinated proteins, and non-specific binding occurs under the mild immunoprecipitation conditions associated with using 10 mM HEPES solution. We enriched polyubiquitinated substrates specifically binding to the UBA domain in hFAF1, using a tandem immunoprecipitation (IP) procedure shown in Fig. 5A. HEK293T cells co-transfected with Flag-hFAF1 and HA-ubiquitin or HA vector, were treated with the proteasome inhibitor, MG132 (10 μM) for 8 h, lysed and the immune complexes containing hFAF1 and interacting polyubiquitinated proteins were purified by incubating with Flag M2 agarose beads. The immune complexes of hFAF1 interacting proteins were solubilized with 1% SDS to dissociate the complex, and diluted 10 fold with buffer not containing SDS. Polyubiquitinated proteins bound to hFAF1 were obtained by a second immunoprecipitation of immune complex with anti-HA antibody crosslinked beads. Polyubiquitinated proteins specifically interacting with UBA domain in hFAF1 were separated on 10% SDS PAGE, and detected with western analysis using anti-HA antibody (Fig. 5B) or silver staining (Fig. 5C) and confirmed with UBA specific immunoprecipitation (Fig. 5B). In the first immunocomplex (input), polyubiquitinated proteins recruited by hFAF1 exist both in HA and HA-ubiquitin samples, and after the second immunoprecipitation (sample) ubiquitinated proteins exist only in HA-ubiquitin overexpressing samples. As shown in Fig. 5B, we could specifically enrich polyubiquitinated proteins interacting with UBA domain in the second immunoprecipitated sample. Polyubiquitinated proteins were separated on 10% SDS gel (Fig. 5C), and the high molecular weight region bands (bands 1–4, >100 KDa) were identified by peptide sequencing using UPLC/nanospray ESI-q-TOF tandem MS. The high molecular weight region bands from the control lane were analyzed similarly and this background was excluded. As we previously reported, hFAF1 mainly interacts with Lys-48 linked polyubiquitin chains (17), and a small fraction (<5%) of polyubiquitin chains binding to hFAF1-UBA domain included a polyubiquitin chain containing Lys63-, Lys29-, Lys6- and Lys11-linked moieties (data not shown). In addition to polyubiquitin chains, we identified Hsp70 in bands 2, 3 and 4 as candidates for UBA domain substrate, representing polyubiquitinated UBA domain binding proteins (Fig. 5C and 5D). Thus tandem immunoprecipitation permitted enriching polyubiquitin chains binding to hFAF1-UBA domain and use of these samples as appropriate substrates for identifying proteins binding to hFAF1-UBA. In addition to Hsp70, several possible polyubiquitinated substrates of hFAF1-UBA were identified in the high molecular weight bands (bands 2, 3 and 4) as shown in Fig 5C. Fez family zinc finger protein 1 (FEZF1)**,** tyrosine-protein phosphatase non-receptor type 14 (PTPN14) were identified in band 2, and polyubiquitinated lysine sites were identified by MS/MS in PTPN14 as shown in Table S1. The proteins binding to UBA domain of hFAF1, could be further studied because of the high abundance of Hsp70 binding.

### Polyubiquitinated Hsp70 binds to hFAF1 through its UBA domain

**Figure 6:**
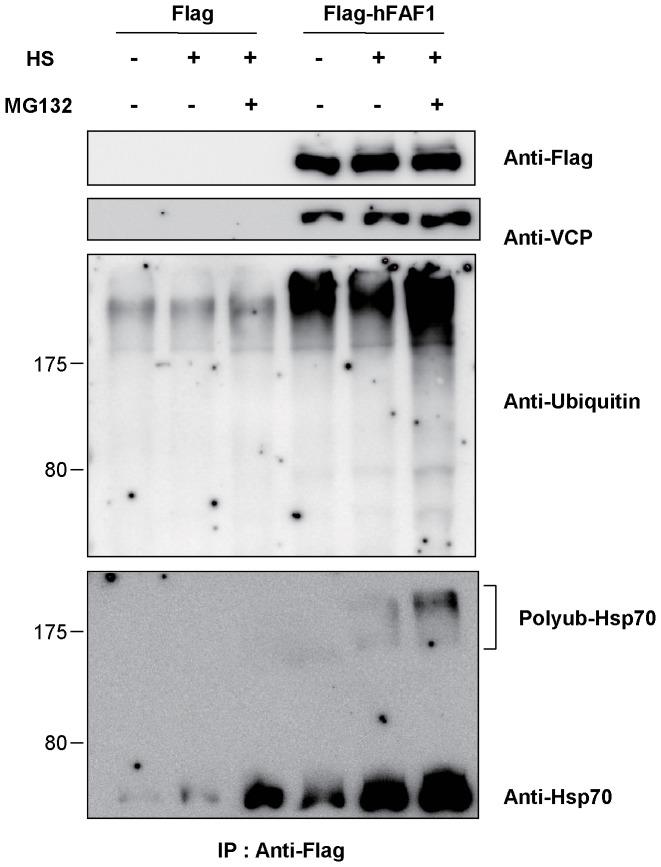
Polyubiquitinated Hsp70 interacts with UBA domain of hFAF1. HEK293T cells were transfected with Flag-CMV2 or Flag-CMV2-hFAF1. 24 h after transfection, cells were heat shocked at 45°C for 45 min, and 12 h after heat shock, treated with MG132 (10 μM) for 8 h. Cells were lysed with 0.5% Triton X-100 hypotonic solution, and lysates were immunoprecipiated with anti-Flag cross-linked agarose beads. Precipitates were separated in 10% SDS PAGE and detected with anti-Flag, anti-VCP, anti-ubiquitin, and anti-Hsp70 antibody.

We examined whether hFAF1 interacts with endogenous polyubiquitinated Hsp70, by examining the interaction employing immunoprecipitation. HEK293T cells overexpressing Flag or Flag-hFAF1 were treated with proteasome inhibitor MG132 (10 μM) for 8 h to enrich polybiquitinated proteins inside cells after heat shock (45°C for 45 min). The cell lysates were immunoprecipitated with monoclonal anti-Flag M2 agarose crosslinking affinity beads and the immune complexes were analyzed by western analysis using anti-Flag, anti-VCP, anti-ubiquitin and anti-Hsp70 antibodies. We detected Hsp70 in the higher molecular weight regions by western blotting (Fig. 6). This suggests that polyubiquitintated Hsp70 bound to hFAF1, accumulated in cells treated with MG132 after heat shock. These results confirm that endogenous Hsp70 was polyubiquitinated and bound to hFAF1 UBA domain.

### hFAF1 promotes Hsp70 degradation

**Figure 7:**
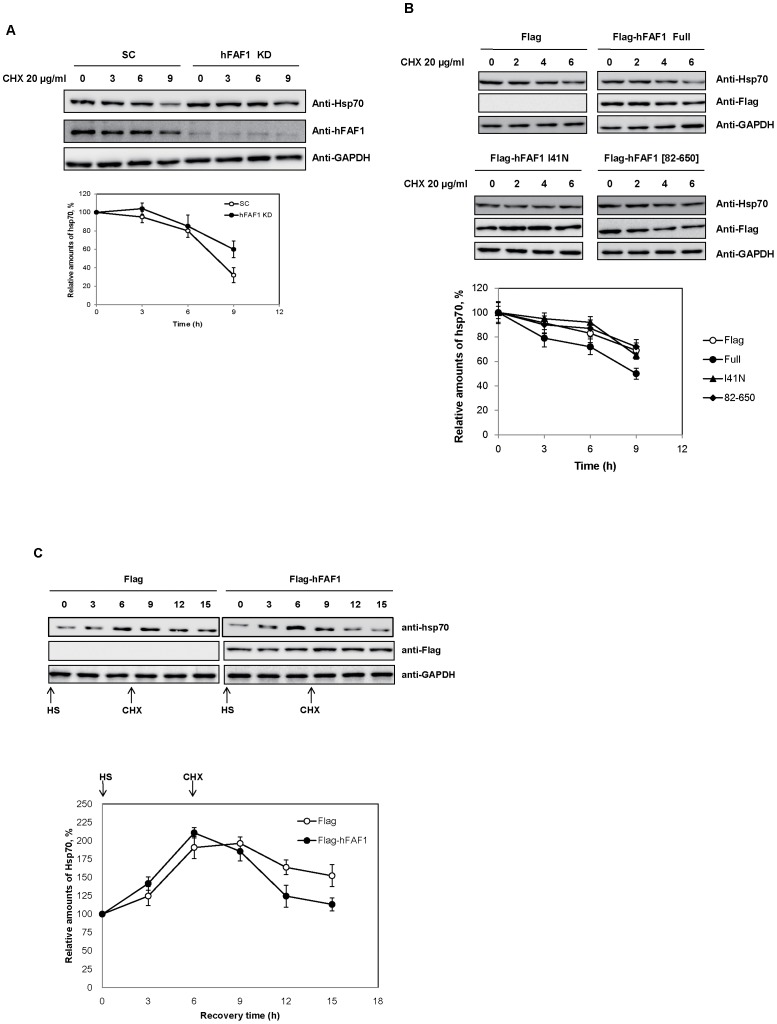
hFAF1 regulates turnover of Hsp70 (**A**) Turnover of hsp70 was measured in hFAF1-knocked down HeLa cells, compared to that in control cells treated 10 μg/mL cycloheximide for indicated times. (**B**) Turnover of Hsp70 was measured in hFAF1 WT and hFAF1 ubiquitin related domain mutants. HeLa cells were transfected with Flag-CMV2 vector, Flag-hFAF1 WT, Flag-hFAF1 [82–650] and Flag-hFAF1 I41N, and treated 10 μg/mL cycloheximide for indicated times. Cells were lysed, separated in 10% SDS PAGE and immunoblotted with Hsp70 and GAPDH antibody. The amounts of Hsp70 remaining after cycloheximide treatment were determined by ImageQuant. (C) Turnover of hsp70 was measured in Flag-hFAF1 overexpressing cells and Flag control vector during heat shock recovery. HeLa cells transfected with Flag or Flag-hFAF1, and 24 h. After transfection, cells were treated heat shock at 45°C for 10 min, and allowed to recover at 37°C for 6 h, and then treated with 20 μg/mL cycloheximide for 9 h. Cells were lysed, separated in 10% SDS PAGE and immunoblotted with hsp70 and GAPDH antibody. The amounts of hsp70 that remained were determined by ImageQuant.

CHIP, an E3 ligase, has been shown to induce K48- and K29-linkage ubiquitination of Hsp70 and regulates Hsp70 degradation during stress response [19]. Parkin induces mono-ubiquitinated Hsp70 and regulates JNK suppression by Hsp70 [10], [21]. We have shown, employing *in vitro* and *in vivo* binding assays and structural studies, that hFAF1 mainly interacts with K-48 linked polyubiquitin chains and not with mono-ubiquitin [9], [17]. Since it is likely that hFAF1 binds to K48-linked polyubiquitinated Hsp70 and regulates the turnover of Hsp70, we examined whether hFAF1 affects the degradation of Hsp70. Cellular Hsp70 turnover was measured in HeLa cells in which hFAF1 was stably knocked down, and in control cells, by treating them with cycloheximide (10 μg/mL), a protein synthesis inhibitor, for various times, and monitoring cellular Hsp70 levels with western analysis using anti-Hsp70 antibody. As shown in Fig. 7A, Hsp70 turnover decreased in hFAF1 knocked down cells, and not in control cells. This indicates that hFAF1 accelerates Hsp70 degradation. This might explain our previous finding [8] that hFAF1 interacts with Hsp70 in UBL1 domain and negatively regulates the chaperone activity of Hsp70.

To confirm that hFAF1 promotes the degradation of Hsp70 by interacting with polyubiquitinated Hsp70 through UBA domain, we measured Hsp70 turnover rates in cells overexpressing full-length hFAF1 and hFAF1 mutants lacking in UBA domain. We employed pFlag-hFAF1 I41N rather than pFlag-hFAF1[1–81], because pFlag-hFAF1 I41N does not bind to polyubiquitins having full hFAF sequence [17]. HEK293T cells transiently transfected with various constructs including pFlag-hFAF1, pFlag-hFAF1[82–650] (UBA domain truncated mutant) or pFlag-hFAF1 I41N, were treated with cycloheximide for various times and cellular Hsp70s monitored as described. As shown in Fig. 7B, cells expressing wild type hFAF1 discernibly promote Hsp70 degradation, while cells expressing hFAF1[82–650] and hFAF1 I41N acted like control cells. These results confirm that polyubiquitinated Hsp70 can be an UBA domain substrate, and that promotion of its degradation by hFAF1 requires wild type UBA domain binding to polyubiquitinated proteins. Since polyubiquitinated Hsp70 accumulates after MG132 treatment following heat shock, we examined whether hFAF1 affects the degradation of heat shock induced Hsp70 during recovery after heat shock. HeLa cells transfected with Flag and Flag-hFAF1 were exposed to heat shock at 45°C for 10 min, and allowed to recover for 6 h at 37°C and then treated with cycloheximide for 9 h. At 6 h of recovery after heat shock, Hsp70 expression increased as shown in Fig. 7C. Hsp70 induced by heat shock in cells overexpressing hFAF1 was more readily degraded by treating with cycloheximide, compared to control cells. These results demonstrate that hFAF1 promotes Hsp70 degradation in both normal and stressed conditions and that this is due to the interaction of polyubiquitinated Hsp70 with UBA domain of hFAF1.

## Discussion

We previously demonstrated that hFAF1 has an UBA domain which acts as an ubiquitin receptor, and recruits polyubiquitinated proteins and stabilizes the ubiquitinated proteasomal substrates [9]. In the present study, we showed that hFAF1's interaction with polyubiquitinated proteins through its UBA domain, causes the suppression of tumor colony formation. We also showed that overexpression of hFAF1 itself causes cancer cell death mediated by the UBA domain. This is the first study that defines the role of hFAF1 in tumor formation, and demonstrates that N-terminal UBA domain of hFAF1 is the responsible moiety. Our examination of the correlation between cervical cancer and hFAF1 expression levels revealed that hFAF1 expression is significantly reduced in cervical cancer tissues (Fig. 1B), confirming a previous report that hFAF1 expression was significantly reduced in 50% of signet ring cells in gastric cancer [7].

To determine whether hFAF1 is a cause or a result of tumorigenesis, we examined the effects of overexpression and knock-down of hFAF1 on tumorigenesis by studying colony formation in soft agar and signaling pathways. We found that hFAF1 significantly inhibits tumor growth, and that this tumor inhibition occurs because the N-terminal UBA domain of hFAF1 interacts with and inhibits the degradation of ubiquitinated proteins. Overexpression of just the UBA domain, increased cell death, and activated JNK and caspase 3, as much as the overexpression of full-length hFAF1 did. Expression of hFAF1[82–650] in which the UBA domain is absent, not only failed to increase cell death, but also actually increased the cell survival (Fig. 3). These results suggest that hFAF1 serves as an inhibitor of tumor formation by regulating protein degradation in the ubiquitin-proteasome pathway via its UBA domain. Further studies characterizing the structures of hFAF1 and its UBA moiety, and the UBA substrates, may help reveal how the UBA domain of hFAF1 causes inhibition of tumor formation. The UBA domain, the first ubiquitin-binding domain described, consisting of approximately 45 residues is found in many proteins of the ubiquitin proteasome system (UPS). UBA domains bind mono- or poly-ubiquitins, and play a wide range of roles mediating cell cycle control, activating DNA repair and promoting proteasomal degradation [14]. The UBA domain consists of three-helical bundles and a highly conserved hydrophobic patch. Over 100 human proteins containing a UBA domain have been identified, including UBP, E2, E3; UV excision repair proteins such as Rad23; protein kinases such as p62 involved in cell signaling pathways, and cell cycle control [15], [22]. Although all the UBA domains have similar structures [23], they have different electronic surface potentials which seem to be specific for recognizing different polyubiquitin linkages and substrates. Evidence indicating that individual UBA domains have different and specific functions, is accumulating [24]. A recent study reported that mammalian UBA domains have diverse polyubiquitin interaction preferences and sensitivity environmental effects [25]. hFAF1 UBA domain also has typical UBA domain structure, including three helices and a hydrophobic residue, but does not have MGF motif, or hydrophobic patch crucial for interacting with ubiquitin. In a previous study, employing NMR spectroscopy, we confirmed binding of hFAF1 N-terminal UBA domain with polyubiquitiated proteins, but not with monoubiquitin, *in vivo*. hFAF1 mainly interacts with K-48 linked ubiquitin chain [9], [17]. It appears that proteasome substrates require specific ubiquitin receptors which recognize ubiquitinated substrates to present to the proteasome [13]. The substrate selectivity of specific receptors remains to be elucidated. To further investigate UBA domain function we employed various UBA substrates and a specific tandem immunoprecipitation method, to enrich the polyubiquitinated proteins and facilitate interaction with UBA domain, and identified polyubiquitinated Hsp70 as a recruiting protein to UBA domain. We examined the effect of hFAF1 on the half life of Hsp70, and found that hFAF1 promotes Hsp70 degradation both in normal and stressed conditions. This was confirmed in cells in which hFAF1 was knocked down. Hsp70 degradation was significantly reduced in cells deficient hFAF1 compared to control cells. We investigated whether the UBA domain of hFAF1 regulates the degradation of Hsp70, employing various mutants of the UBA domain, e.g. hFAF1[82–650] and hFAF1 I41N, mutant in which UBA domain is deleted, and hFAF1 I41N, a mutant defective in binding to polyubiquitinate proteins [17]. Both these mutants of hFAF1, lack the ability of hFAF1 to accelerate the degradation of Hsp70 and cause the accumulation of undegraded Hsp70. This study suggested that Hsp70 bound to UBL1 is rapidly ubiquitinated, and that the ubiquitinated Hsp70 can bind to UBA domain to be eventually degraded in the proteasome. We previously showed that hFAF1 negatively regulates Hsp70's chaperone activity by promoting the interaction of hFAF1 UBL1 domain with Hsp70 [8]. We did not explain how. We now think that hFAF1 can enhance the degradation of Hsp70 as an ubiquitin receptor, and negatively regulate the chaperone activity of Hsp70. Polyubiquitinated Hsp70 [26], and several E3 ligases are known to ubiquitinate Hsp70. Carboxyl terminus of Hsp70 binding protein (CHIP) has dual functions as a co-chaperone and a ubiquitin ligase, which target chaperone substrates for proteasomal degradation through Hsp70 binding. In a recent study, CHIP was shown to ubiquitinate both Hsp70 and Hsc70, via K-48, K-29 or K-63 linkage ubiquitin chains [27], and alter the half life of Hsp70 during recovery after stress [19]. CHIP is also involved in multiple steps of the stress response and Hsp70 induction, by activating HSF-dependent and HSF-independent mechanisms. These findings suggest that hFAF1 binds to Hsp70 and ubiqutinated Hsp70s, and regulates Hsp70 protein levels (Fig. 6). Parkin, another E3 ligase containing RING domain, was reported to monoubiquitinate Hsp70 at multiple sites, even without promoting the degradation of Hsp70 [20], [21]. However, complexes of hFAF1-chaperone with CHIP or Parkin, were not detected in this study (data not shown). hFAF1 is significantly down-regulated in cervical cancer tissues. In contrast, Hsp70 is highly overexpressed in these tumor tissues, a common feature in human tumors, that results from the adaptation of cancer cells to adverse conditions [18].

The ubiquitin receptor p62, has been extensively studied in the context of the relationship between UBA domain and disease. Mutation in UBA domain of p62 is implicated in Paget Disease of the Bone (PDB), a genetic disorder of aberrant osteoclastogenic activity [28], [29]. This mutation abolishes TRAF6 polyubiquitination and suggests a possible mechanism in which p62 regulates the NF-κB pathway [22]. The present study shows that UBA domain in hFAF1 is responsible for cancer cell death and colony formation through regulation of Hsp70 degradation, revealing yet another function of UBA domain related to cancer.

Many proteins in ubiquitin pathway are known to act as proto-oncogenes and tumor suppressors. For instance, deubiquitinating enzyme, CYLD (Cylindromatosis tumor suppressor gene) is believed to suppress cell proliferation and tumorigenesis by removing K63-linked ubiquitin chains from TRAF2 and IKKγ thereby inhibiting NF-κB activation [30]. BRCA1 (breast cancer tumor suppressor 1) is a RING finger-containing ubiquitin ligase of importance to breast cancer [31]. Complexation of BRCA1 with another RING finger ubiquitin ligase called BARD1 (BRCA associated RING domain 1) generates a potent ubiquitin ligase that impacts transcription, DNA repair and response to DNA damage [32], [33]. Proteasome inhibitors have been found to possess antitumor activity and are incorporated into the cancer therapy regimens. The validity of targeting the UPS for inhibition of cancer is apparent from the success of this approach in various cancers [34]. Based on the present study, we propose that hFAF1 is a candidate tumor suppressor that operates through its ubiquitin receptor function.

To identify the relevant specific function of UBA domain in hFAF1, we employed UBA substrates and specific immunoprecipitation (Fig. 5). This is a tandem immunoprecipitation technique to enrich polyubiquitinated proteins bound to hFAF1. In addition to Hsp70, we identified β-catenin, Fez family zinc finger protein 1 (FEZF1), tyrosine-protein phosphatase non-receptor type 14 (PTPN14) as candidate UBA substrates. This suggests that hFAF1 plays a role in kinase signaling, and this should be further explored.

Recently, hFAF1 was reported to suppress NF-κB activity by interfering with nuclear translocation of RelA subunit of NF-κB and also to inhibit IκB kinase activation [4], [5]. A homolog of hFAF1 observed in *Drosophila*, called *Caspar*, was also found to inhibit NF-κB. Caspar negatively regulates the immune deficiency (Imd)-mediated responses by blocking nuclear translocation of NF-κB [35]. However, further studies are needed to define the relationship between the inhibition of NF-κB and UBA domain effect. If E3 ligases interact with hFAF1, this could be another way to elucidate the role of hFAF1 as an ubiquitin receptor. Recently UBX domain containing proteins were reported to interact with various E3 ligases. For example, hFAF1 interacts with Cullins and UBRs [36]. E3 ligase based strategy is therefore another way to elucidate ubiquitin receptor function of hFAF1.

In summary, our studies demonstrate that hFAF1 plays a key role in tumor formation in cervical cancer by recruiting polyubiquitinated proteins through its UBA domain. A reciprocal relationship exists between expression level of hFAF1 and cervical cancer. We identified that polyubiquitinated Hsp70 is an ubiquitin substrate of UBA domain of ubiquitin receptor hFAF1 by employing tandem immunoprecipitation, and demonstrated that hFAF1 negatively regulates chaperone activity of Hsp70 by promoting the degradation in proteasome via UBA domain.

## Materials and Methods

### Chemicals, antibodies, and constructs

pFlag-CMV-2-hFAF1 full, pFlag-CMV-2-FAF1[1–81], pFlag-CMV-2-FAF1[82–650] [9], pFlag-CMV-2-hFAF1 I41N were prepared as previously described [17]. Polyclonal anti-serums against hFAF1 were generated in rabbits using recombinant human hFAF1 [1–81]. The sources of other reagents were as follows: Monoclonal anti-Hsc70/Hsp70 antibody was purchased from Assay designs Inc. (MI, USA); Monoclonal anti-HA and polyclonal anti-GAPDH antibody from AbFrontier (Seoul, Korea); Monoclonal anti-Flag antibody (M2) from Sigma (MO, USA); anti-VCP from Fitzgerald Industries (MA, USA), anti-caspase3 from BD Biosciences (KY, USA), anti-ubiquitin antibody from Chemicon International (CA, USA), anti-PARP and anti-JNK and anti-phospho-JNK (Thr183/Tyr185) from Santa Cruz Biotechnology (CA, USA), and anti-Akt and anti-phospho-Akt (Ser473) from Cell Signaling Technology (MA, USA); the proteasome inhibitor MG132 from A.G. Scientific, Inc. (CA, USA), and protein synthesis inhibitor cycloheximide and MTT assay kit from Sigma.

### Cell culture and transient transfection

HeLa cells were grown and maintained in MEM. HEK293T cells were grown in DMEM supplemented with 10% fetal bovine serum and 1% penicillin. The cells were maintained in a constant atmosphere of 5% CO_2_ at 37°C. They were transfected with expression plasmids using Lipofectamin (Invitrogen, USA) or Effectene (Qiagen, Germany) according to manufacturer's recommendations. Cells were routinely examined and used 24 h after transfection.

### Knocking-down hFAF1 in the cell line

Lentiviral shRNA vectors for targeting hFAF1 gene were constructed by inserting synthetic double stranded oligonucleotides into the shLenti1.1 lentiviral vector (Macrogen, Seoul, Korea). The target sequence is 5′-GGAAGACAGTACGGTTCTAAA-3′ for #1, 5′-GGAACAGTCGGAAGAACAAAT-3′ for #2 and 5′-AAGCTCCAGATTGTCTTTGATTT-3′ for #3. The shLenti1.1 was designed to produce shRNAs promoted from U6 promoter and to express LacZ- puromycin fusion protein from hCMV promoter. As a control vector, scrambled sequence 5′-AATCGCATAGCGTATGCCGTT-3′ was inserted into the shLenti1.1 lentiviral vector. Cells infected with lenti-viral hFAF1 shRNA were selected with puromycin (0.4 μg/mL) for one week. Cells expressing lenti-viral hFAF1 shRNA were monitored by X-gal staining. Cells infected with lenti-virus were fixed for 5 min in a fixing solution (1% formaldehyde and 0.2% glutaraldehyde), stained with X-gal staining solution (4 mM potassium ferrocyanide, 2 mM MgCl_2,_ 0.4 mg/mL X-gal in PBS) overnight at 37°C and examined by light microscopy.

### Colony formation assay

Soft agar colony formation assay was performed after seeding cells in a layer of 0.35% agarose MEM/FBS layered over 0.5% agarose/MED/FBS. Agarose bottom was prepared, by mixing sterilized 1% agarose with the same volume of MEM medium containing 10% FBS and solidified for 30 min at room temperature. When the bottom agarose completely solidified, transfected cells were harvested and counted. After mixing sterilized 0.7% top agarose and 10^6^ cells/mL in 1∶1 ratio, the liquid agarose containing the cells was solidified at room temperature, and then incubated for about 2∼3 weeks at 37°C until colony formation. The colonies were counted and data presented as ± SD. All measurements were done at least in triplicate.

### Cell Survival Assay

Cell viability was monitored by MTT assay and FACS analysis. For MTT conversion assay, HeLa cells transiently transfected with the expression plasmid using Lipofectamine were seeded in 96 well plates at a density of 1 x 10^5^ cells/well, and incubated for indicated times (24, 48 and 72 h). Ten μL of MTT (5 mg/mL, Sigma) was added, and incubation continued for 2 h at 37°C to allow conversion of MTT to 3-[4,5-dimethyldiazol-2-yl]-2,5-diphenylformazan. The purple precipitate was solubilized in 100 μL acidified isopropanol and absorbance was measured at 570 nm using a micro plate reader (Softmax Pro5, USA). For FACS analysis, the cells were fixed in 70% ethanol overnight at 4°C and stained with PI solution containing 50 μg/mL propidium iodide (Sigma, USA) and 200 μg/mL RNase (Sigma, USA) for 3 h at 37°C. The stained cells were filtered in a Spectra nylon mesh (Spectrum, 100 μm pore size, USA) and 10,000 cells were measured with a FACS flow cytometer (Beckton & Dickinson). The numbers of apoptotic cells were determined using Mod-fit (Cellquest Software). All experiments were conducted at least in triplicates.

### Immunoblot Analysis

For western analysis, proteins (30 μg) were separated on SDS-PAGE under reducing conditions, transferred to PVDF membrane, and probed with various antibodies. The protein-antibody complexes were visualized after treatment with 1∶2000 diluted HRP (horseradish peroxidase)–conjugated secondary antibody (Cell Signaling Technology, USA) for 1 h. The blots were incubated for 3 min in ECL plus kit (GE Healthcare, UK) and exposed to LAS3000 (Fuji photo film Co., Japan).

### Extraction of Proteins from cancer tissues

Human cervical cancer tissues were obtained by the Department of Pathology, Samsung Medical Center, Sungkyunkwan University School of Medicine, Seoul, Korea, from patients who gave written informed consent. All samples were collected according to the Institutional Review Board Guidelines. The institutional review board of our hospital approved this study (IRB No. 2006-08-095). The tissues were immediately snap-frozen at −80°C, washed three times with cold PBS and chopped with a sterilized surgical lancet. Red blood cells (RBCs) were removed in RBC lysis buffer. Tissue segments were homogenized in 5 times the tissue volume of a lysis buffer (10 mM Tris-acetate, pH 7.4, 10 mM NaCl, 0.1 mM EDTA, protease inhibitors), centrifuged for 30 min at 1200 rpm at 4°C, and the proteins in the supernatant were quantified by Bradford assay.

### UBA-specific tandem immunoprecipitation

HEK293T cells were co-transfected with Flag-hFAF1 and HA-ubiquitin or HA-vector as a control and treated with 10 μM MG132 for 8 h to accumulate polyubiquitinated proteins. In the first step, cells were lysed with a lysis buffer containing protease inhibitors (10 mM HEPES, 1.5 mM MgCl_2_, 10 mM KCl, 0.5% NP-40, 0.5 mM PMSF, 5 μg/mL aprotinin, 10 μg/mL pepstatin A, 10 μg/mL leupeptin, pH 7.9) for 30 min on ice. The cell lysates were immunoprecipitated with anti-Flag M2 agarose-linked affinity beads. The lysates were centrifuged at 14,000 x g for 15 min, and the supernatants were incubated with monoclonal anti-Flag M2 agarose cross-linking affinity beads (Sigma, USA) at 4°C for 3 h. Precipitates were eluted with a buffer containing 100 mM Tris-Cl, pH 7.5, and 1% SDS and were diluted (10 x) with an IP buffer (50 mM Tris-Cl, 150 mM NaCl 0.5% NP-40, pH 7.5) to reduce the SDS to 0.1%. In the second step, the diluted supernatants were incubated with HA affinity beads at room temperature. The beads were washed with second IP buffer and the immune complexes were separated on SDS-PAGE and detected by silver staining or western analysis. Protein bands on silver stained gels were identified by amino acid sequencing using UPLC-ESI-q-TOF tandem MS.

### Mass spectrometric analysis of polyubiquitin

The gel bands were destained and digested with trypsin and the resulting peptides extracted as previously described [37], evaporated to dryness in SpeedVac for MS analysis, dissolved in 10% ACN solution containing 1.0% formic acid, and desalted on line prior to separation using trap column (5 µm particle size, NanoEase^TM^ dC18, Waters) cartridge. Peptides were separated using a C18 reverse-phase 75 µm i. d. x 150 mm analytical column (3 µm particle size, Atlantis^TM^ dC18, Waters) with an integrated electrospray ionization SilicaTip^TM^ (± 10 µm i.d., New Objective, USA). Chromatography was performed on line to mass spectrometer (Q-tof Ultima^TM^ global, Waters Co. UK).

Raw data files obtained from the mass spectrometer were converted to. pkl files using ProteinLynx Global Server^TM^ (PLGS) 2.3 data processing software (Waters Co. UK). The MS/MS spectra were matched against amino acid sequences in SwissProt. Large numbers and types of potential PTMs were considered. All reported assignments were verified by both automatic and manual interpretation of spectra from Mascot and MOD^i^ (Korea, http://prex.uos.ac.kr/modi/) in a blind mode [38].

### Immunoprecipitation

Cells were disrupted with a lysis buffer containing protease inhibitors (10 mM HEPES, 1.5 mM MgCl_2_, 10 mM KCl, 0.5% NP-40, 0.5 mM PMSF, 5 μg/mL aprotinin, 10 μg/mL pepstatin A, 10 μg/mL leupeptin, pH 7.9) for 30 min on ice. The lysates were centrifuged at 14,000 x g for 15 min, and the supernatants were incubated with monoclonal anti-Flag M2 agarose cross-linking affinity beads (Sigma) at 4°C for 3 h. The beads were washed by gradient washing buffer containing 0.5% NP40. After first washing with lysis buffer, the washing buffer concentration was increased gradually up to 50 mM Tris-Cl, 150 mM NaCl.

## Supporting Information

Table S1 **List of UBA substrates in hFAF1 identified by UPLC/nano ESI-q-TOF tandem MS.**
(PPT)
