# Supplementary material for: Ubiquitin-Associated (UBA) Domain in Human Fas Associated Factor 1 Inhibits Tumor Formation by Promoting Hsp70 Degradation
Source: PLoS One. 2012 Aug 2;7(8):e40361. doi: 10.1371/journal.pone.0040361 (PMC3410879; doi:10.1371/journal.pone.0040361)
Supplement: Table S1 — List of UBA substrates in hFAF1 identified by UPLC/nano ESI-q-TOF tandem MS. (PPT) [file pone.0040361.s001.ppt]

## Slide 1
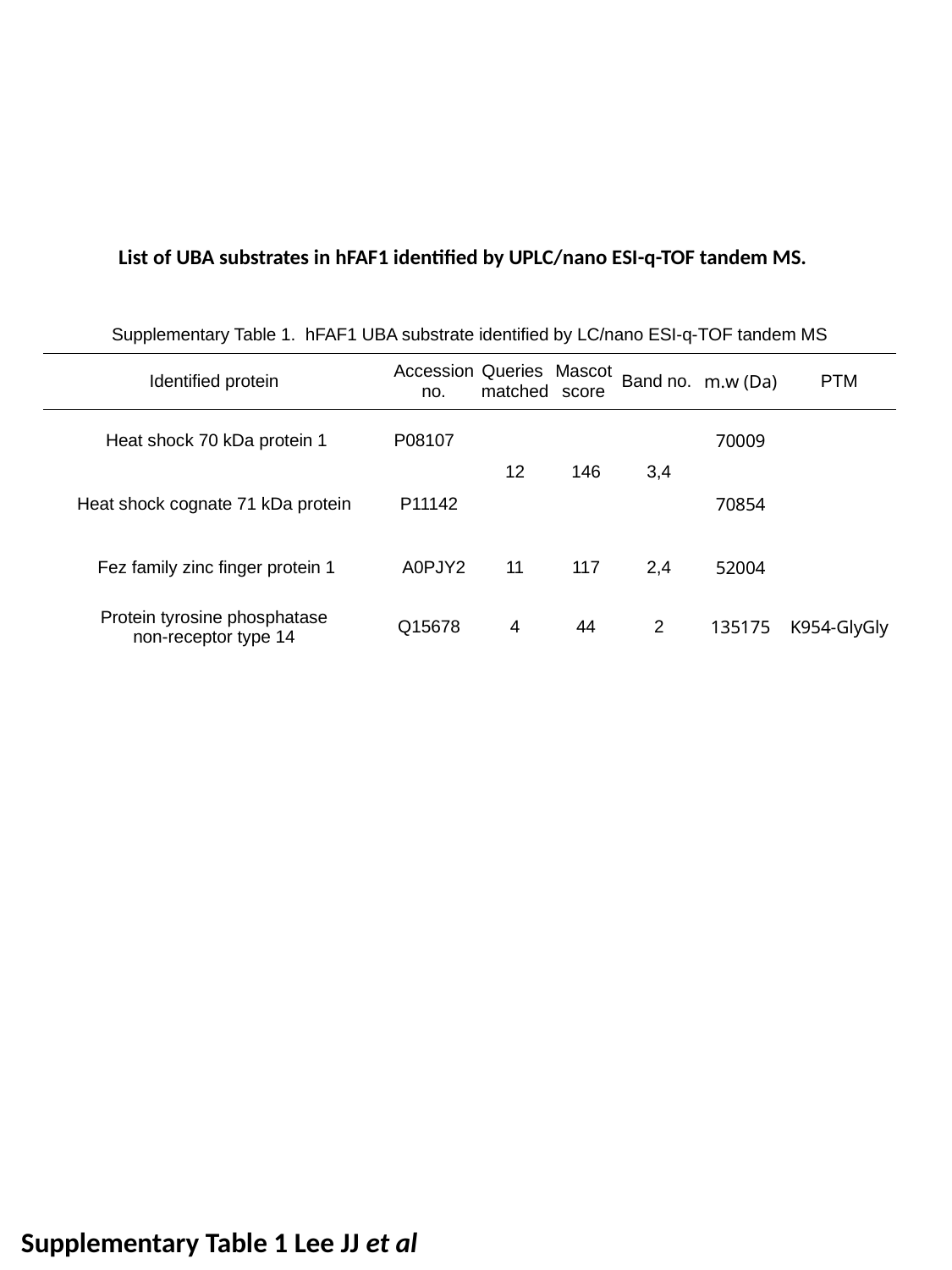

List of UBA substrates in hFAF1 identified by UPLC/nano ESI-q-TOF tandem MS.
| Supplementary Table 1. hFAF1 UBA substrate identified by LC/nano ESI-q-TOF tandem MS | | | | | | |
| --- | --- | --- | --- | --- | --- | --- |
| Identified protein | Accession no. | Queries matched | Mascot score | Band no. | m.w (Da) | PTM |
| Heat shock 70 kDa protein 1 | P08107 | 12 | 146 | 3,4 | 70009 | |
| Heat shock cognate 71 kDa protein | P11142 | | | | 70854 | |
| Fez family zinc finger protein 1 | A0PJY2 | 11 | 117 | 2,4 | 52004 | |
| Protein tyrosine phosphatase non-receptor type 14 | Q15678 | 4 | 44 | 2 | 135175 | K954-GlyGly |
Supplementary Table 1 Lee JJ et al
